# Another look at the eigenvalues of a population matrix model

**DOI:** 10.7717/peerj.8018

**Published:** 2019-11-11

**Authors:** Brenda Hanley, Patrick Connelly, Brian Dennis

**Affiliations:** 1 Cornell Wildlife Health Lab, Department of Population Medicine and Diagnostic Sciences, Cornell University, Ithaca, NY, United States of America; 2 Department of Statistical Sciences, University of Idaho, Moscow, ID, United States of America; 3 Department of Fish and Wildlife Sciences, University of Idaho, Moscow, ID, United States of America

**Keywords:** Balance equation, Characteristic equation, Projection matrix, Asymptotic growth rate, Dominant eigenvalue, Leslie matrix, Lefkovitch matrix, Interactive software, Wildlife

## Abstract

Population matrix models are important tools in resource management, in part because they are used to calculate the finite rate of growth (“dominant eigenvalue”). But understanding how a population matrix model converts life history traits into the finite rate of growth can be tricky. We introduce interactive software (“IsoPOPd”) that uses the characteristic equation to display how vital rates (survival and fertility) contribute to the finite rate of growth. Higher-order interactions among vital rates complicate the linkage between a management intervention and a population’s growth rate. We illustrate the use of the software for investigating the consequences of three management interventions in a 3-stage model of white-tailed deer (*Odocoileus virginianus*). The software is applicable to any species with 2- or 3-stages, but the mathematical concepts underlying the software are applicable to a population matrix model of any size. The IsoPOPd software is available at: https://cwhl.vet.cornell.edu/tools/isopopd.

## Introduction

Population matrix models (PMMs) are used to assess strategies to manage populations for resource purposes. These models are regularly found in quantitative ecology texts (e.g., Fryxell, Sinclair & Caughley, 2014), and have been used in many applied settings (Salguero-Gómez et al., 2014; Salguero-Gómez et al., 2016). But how familiar are broad audiences with the mathematics behind the PMM, particularly those that generate the most prominent model output, the finite rate of growth? We use a visual approach to provide an alternative way to understand how targeted managerial alterations to a life cycle can alter the finite rate of growth.

The life history traits of a species (“vital rates”) govern the mathematical structure of the PMM (De Kroon, Van Groenendael & Ehrlen, 2000). The vital rates include stage-wise fertilities, survival, and transition probabilities (Caswell, 2001). The population-scale demographic properties obtained from the PMM include stage abundances, the finite rate of growth (hereafter “ *λ*”), the stable stage distribution, net reproductive values, sensitivities and elasticities (Caswell, 2001), plus a host of transient quantities (Stott, Hodgson & Townley, 2012). For example, let an arbitrary 3-stage PMM be:

(1) \begin{eqnarray*}\mathbf{A}= \left[ \begin{array}{@{}ccc@{}} \displaystyle {A}_{11}&\displaystyle {A}_{12}&\displaystyle {A}_{13}\\ \displaystyle {A}_{21}&\displaystyle {A}_{22}&\displaystyle {A}_{23}\\ \displaystyle {A}_{31}&\displaystyle {A}_{32}&\displaystyle {A}_{33} \end{array} \right] ,\end{eqnarray*}

which contains matrix elements *A*_*ij*_ (*i* = 1, 2, 3, and *j* = 1, 2, 3) and where *A*_1*j*_ is the average fertility in the *j*th stage, *A*_*ii*_ is average survival of the *i*th stage without transition out of the *i*th stage, and *A*_*ij*_ is the average survival of the *j*th stage with transition from the *j*th stage to the *i*th stage. Stage-wise abundances are projected through time using (Caswell, 2001):

(2) \begin{eqnarray*} \left[ \begin{array}{@{}c@{}} \displaystyle {J}_{1}\\ \displaystyle {S}_{1}\\ \displaystyle {B}_{1} \end{array} \right] =\mathbf{A} \left[ \begin{array}{@{}c@{}} \displaystyle {J}_{0}\\ \displaystyle {S}_{0}\\ \displaystyle {B}_{0} \end{array} \right] ,\end{eqnarray*}

where *J*_0_, *S*_0_, and *B*_0_ are the number of stage one, stage two, and stage three individuals at time 0, and *J*_1_, *S*_1_, and *B*_1_ are the number of stage one, stage two, and stage three individuals at time 1. Recursive calculation of Eq. (2) from time 0 through time *n* yields:

(3) \begin{eqnarray*} \left[ \begin{array}{@{}c@{}} \displaystyle {J}_{n}\\ \displaystyle {S}_{n}\\ \displaystyle {B}_{n} \end{array} \right] ={\mathbf{A}}^{n} \left[ \begin{array}{@{}c@{}} \displaystyle {J}_{0}\\ \displaystyle {S}_{0}\\ \displaystyle {B}_{0} \end{array} \right] .\end{eqnarray*}

As *n* gets large, *A*^*n*^ behaves like a single number, denoted *λ* (Caswell, 2001). Depending on the citation, *λ* is called the “dominant eigenvalue”, the “intrinsic rate of growth”, the “asymptotic growth rate”, or simply “the growth rate”. Regardless of which name is chosen, the *λ* is the geometric rate of population change once stage oscillations damp out and the stages approach stable proportions. In other words (Caswell, 2001):

(4) \begin{eqnarray*}Aw=\lambda w,\end{eqnarray*}

where *w* gives the stable stage proportions.

But to broader audiences, Eqs. (1)–(4) do not necessarily clarify the link between cause and effect in population dynamics, especially as they pertain to decision making towards a resource goal. Herein, we attempt to clarify the nature of *λ* as it pertains to strategic planning of targeted managerial interventions in resource management settings.

We introduce the interactive software (“IsoPOPd”) that represents *λ* in a visual framework. The graphics provide an alternative way to think about how managerial alterations to matrix elements (hereafter “*A*_*ij*_”) produce changes in *λ*. We illustrate the use of the software with white-tailed deer (*Odocoileus virginianus*) but remark that the software may be used to understand cause and effect in any 2- or 3-stage PMM. The software is at: https://cwhl.vet.cornell.edu/tools/isopopd.

## Methods

A common life history contains three stages that we define as the juvenile (non-reproducing individual), early adult (with reproduction rates consistent with mid-life fertility), and late adult (with reproduction rates consistent with late-life fertility) stages. We use *A* to represent the PMM for such a species. The characteristic equation for *A* is found in linear algebra textbooks (e.g., Strang, 2016):

\begin{eqnarray*}& & {\lambda }^{3}+(-{A}_{11}-{A}_{22}-{A}_{33}){\lambda }^{2}+({A}_{11}{A}_{22}+{A}_{22}{A}_{33}+{A}_{11}{A}_{33}-{A}_{32}{A}_{23}-{A}_{21}{A}_{12}-{A}_{13}{A}_{31})\lambda \end{eqnarray*}

(5) \begin{eqnarray*}& & +({A}_{11}{A}_{32}{A}_{23}+{A}_{12}{A}_{21}{A}_{33}+{A}_{31}{A}_{13}{A}_{22}-{A}_{11}{A}_{22}{A}_{33}-{A}_{21}{A}_{13}{A}_{32}-{A}_{31}{A}_{12}{A}_{23})=0.\end{eqnarray*}

Using the notation for the superparameters (“coefficients of the characteristic equation”) in Hanley & Dennis (2019), the superparameters are:

(6) \begin{eqnarray*}& & p=(-{A}_{11}-{A}_{22}-{A}_{33}),\end{eqnarray*}

(7) \begin{eqnarray*}& & q=({A}_{11}{A}_{22}+{A}_{22}{A}_{33}+{A}_{11}{A}_{33}-{A}_{32}{A}_{23}-{A}_{21}{A}_{12}-{A}_{13}{A}_{31}),\end{eqnarray*}

and

(8) \begin{eqnarray*}r=({A}_{11}{A}_{32}{A}_{23}+{A}_{12}{A}_{21}{A}_{33}+{A}_{31}{A}_{13}{A}_{22}-{A}_{11}{A}_{22}{A}_{33}-{A}_{21}{A}_{13}{A}_{32}-{A}_{31}{A}_{12}{A}_{23}),\end{eqnarray*}

yielding an abbreviated form of the characteristic equation:

(9) \begin{eqnarray*}{\lambda }^{3}+p{\lambda }^{2}+q\lambda +r=0.\end{eqnarray*}

The software focuses on the link between *p*, *q*, and *r* and *λ*. In particlar, the characteristic equation (Eq. (9)) contains four 3-dimensional volumes (“orthotopes”) that must always balance positive and negative contributions of *p*, *q*, and *r* to zero. In the context of resource management, targeted intervention activities modify the values of *p*, *q*, or *r*, leaving *λ* to respond in a manner that maintains the overall equality. Indeed, Eq. (9) is the set of rules by which *λ* must respond to any managerial strategy. Positive and negative influences on *λ* appear in Table 1.

IsoPOPd was developed to provide an alternative way to understand how *λ* responds to managerial alterations to *p*, *q*, and *r*. The IsoPOPd software converts any PMM into a visual representation of their *p*, *q*, and *r* values, from which *λ* is obtained. The user defines the structure of a 3-stage PMM, then designates the values of each of the nine *A*_*ij*_s. The *Characteristic equation* tab displays the inputs (the PMM itself), the output (the corresponding *λ*), and the mathematical linkage between the two (Eq. (9)). The remaining tabs use a visual framework to illustrate the nature of the linkage. The *Breakdown of the λ*^3^ *term* tab shows the first orthotope, *λ*^3^, a 3-D cube scaled to dimensions (*λ* ×*λ* × *λ*). The *Breakdown of the pλ*^2^ *term* tab shows the second orthotope, *pλ*^2^, the 3-D volume scaled on one side by the vital rates in *p* (Eq. (6)), and on the remaining two sides by *λ*. The negative sign in front of *pλ*^2^ (and the light blue shading in the software graphics) represents a negative contribution to Eq. (9), but an opposite (positive) contribution to *λ*. The *Breakdown of the qλ term* tab shows *qλ*, the trio of sub-orthotopes whose dimensions that may be better understood by rearranging Eq. (7):

(10) \begin{eqnarray*}q=({A}_{11}{A}_{22}-{A}_{21}{A}_{12})+({A}_{22}{A}_{33}-{A}_{32}{A}_{23})+({A}_{11}{A}_{33}-{A}_{13}{A}_{31}).\end{eqnarray*}

The trio of sub-orthotopes in *qλ* are scaled on two sides by the vital rates in Eq. (10) and scaled on the third side by *λ*. A positive sign (and the black shading in the software graphics) represents a positive contribution to Eq. (9), but an opposite (negative) contribution to the final value of *λ*, while a negative sign (and the light blue shading in the software graphics) represents a negative contribution to Eq. (9), but an opposite (positive) contribution to *λ*. The sub-orthotopes in Eq. (10) therefore make positive and negative contributions to *q*, but only the net change is reflected in the final value of *qλ*. Finally, the *Breakdown of the rterm* tab shows the last orthotope, *r*, the 3-D volume that is scaled on all three sides by vital rates. The *Geometric interpretation of the characteristic equation* tab reveals the entire set of graphical orthotopes (*λ*^3^, *pλ*^2^, *qλ*, and *r*) that comprise Eq. (9). Black and light blue shadings in these volumes must always balance.

**Table 1:** Directional contributions of matrix elements to the characteristic equation and *λ*. In the characteristic equation, *λ* is the only term that is free to vary and must do so in a manner that balances all contributions to zero.

Superparameter	Orthotope	Negative contributions to Eq. (9), but positive contributions to *λ*	Positive contributions to Eq. (9), but negative contributions to *λ*
	*λ*^3^	This orthotope shows the response of *λ* to the changes in parameters.	
*p*; Eq. (6)	*pλ*^2^	(*A*_11_)*λ*^2^ (*A*_22_)*λ*^2^ (*A*_33_)*λ*^2^	
*q*; Eq. (7)	*qλ*	(*A*_32_*A*_23_)*λ* (*A*_21_*A*_12_)*λ* (*A*_13_*A*_31_)*λ*	$ \left( {A}_{11}{A}_{22} \right) \lambda $ (*A*_22_*A*_33_)*λ* (*A*_11_*A*_33_)*λ*
*r*; Eq. (8)	*r*	*A*_11_*A*_22_*A*_33_ *A*_21_*A*_13_*A*_32_ *A*_31_*A*_12_*A*_23_	*A*_11_*A*_32_*A*_23_ *A*_12_*A*_21_*A*_33_ *A*_31_*A*_13_*A*_22_

We use the IsoPOPd software to demonstrate the effects on *λ* from hypothetical managerial interventions in white-tailed deer (*Odocoileus virginianus)*. We assume *A* tracks a single sex (female), the *A*_*ij*_ are static, the probability of transition in each stage is one, and each *A*_*ij*_ constitutes an average for members of the *j*th stage (Caswell, 2001). Let the PMM be (Chitwood et al., 2015), reduced to two digits):

(11) \begin{eqnarray*}A= \left[ \begin{array}{@{}ccc@{}} \displaystyle 0&\displaystyle 0.58&\displaystyle 0.70\\ \displaystyle 0.14&\displaystyle 0&\displaystyle 0\\ \displaystyle 0&\displaystyle 0.77&\displaystyle 0.80 \end{array} \right] .\end{eqnarray*}

We investigated how a 10% increase in survival in the 2nd stage (*A*_32_) alters the configuration of the orthotopes in Eq. (9), and ultimately, *λ*. We then investigated how a 10% increase in survival in the 3rd stage (*A*_33_) alters the orthotopes and *λ*. Finally, we investigated a combination scenario where survival is increased by 10% in both 2nd and 3rd stages.

## Results

The characteristic equation for the unperturbed deer scenario (Eq. (11)) is:

(12) \begin{eqnarray*}{\lambda }^{3}+(-{A}_{33}){\lambda }^{2}+(-{A}_{21}{A}_{12})\lambda +({A}_{21}{A}_{12}{A}_{33}-{A}_{21}{A}_{13}{A}_{32})=0,\end{eqnarray*}

or equivalently,

(13) \begin{eqnarray*}{\lambda }^{3}+(-0.8){\lambda }^{2}+(-0.081)\lambda +(-0.0105)=0.\end{eqnarray*}

**Figure 1:**
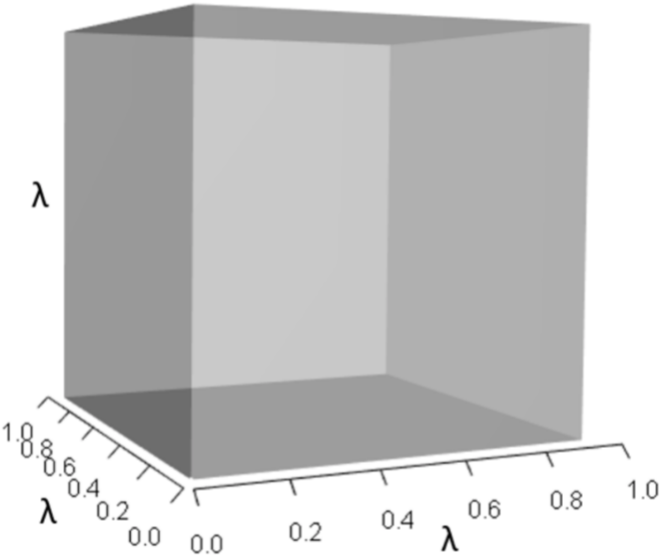
The orthotope of *λ*^3^ in the non-perturbed white-tailed deer scenario (Eq. (13)). Here *λ* = 0.09.

**Figure 2:**
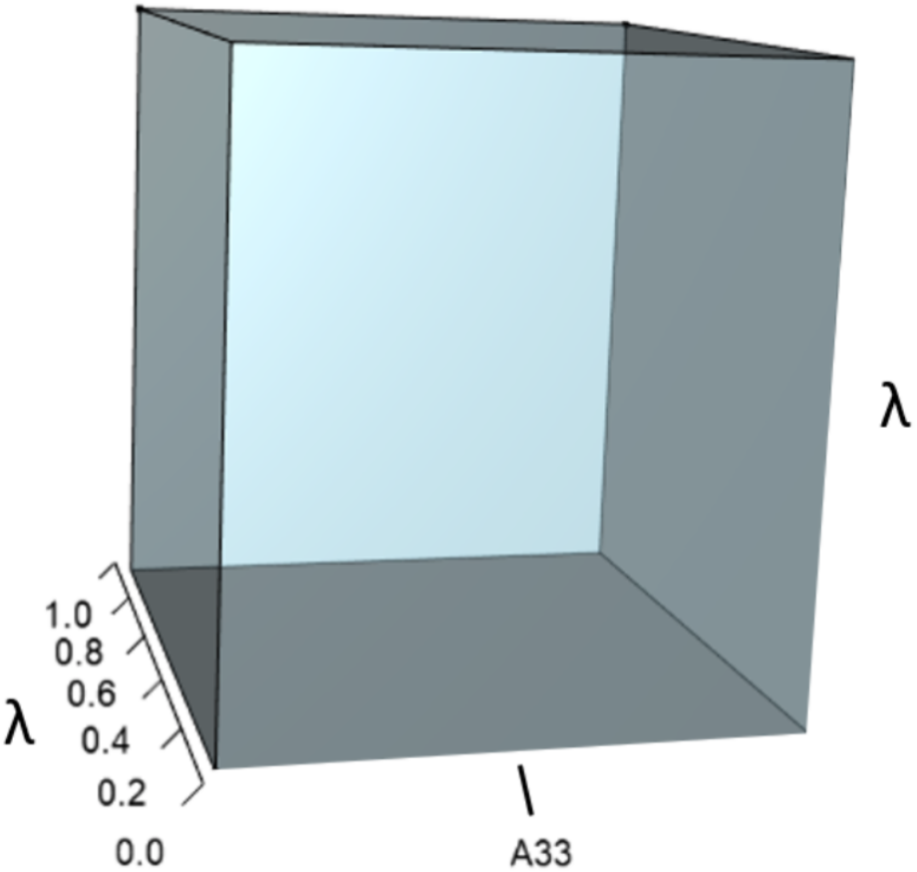
The orthotope of *pλ*^2^ (Eq. (12)). This volume is a negative contribution to Eq. (12) but a positive contribution to *λ* (Table 1).

**Figure 3:**
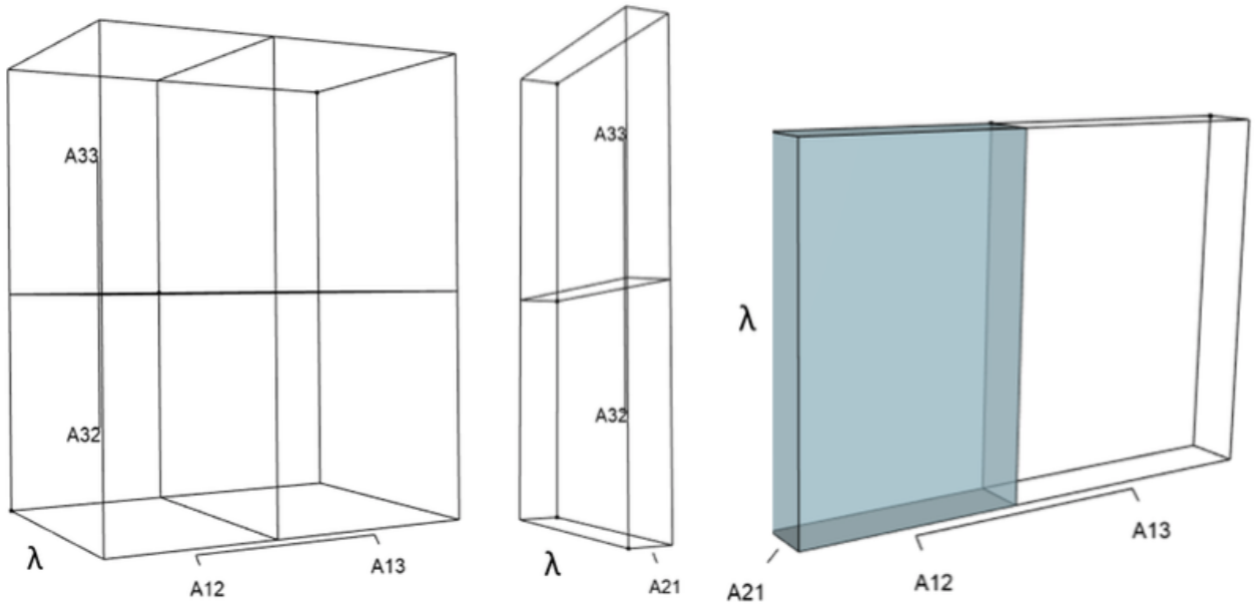
The trio of orthotopes representing the *qλ* term (Eq. (12)). Two dimensions of each orthotope are specified by quadratic interactions of pairwise sets of vital rates, while the third dimension is specified by *λ*. The light blue volume is a negative contribution to Eq. (12), but a positive contribution to *λ* (Table 1). Should management increase *A*_12_ or *A*_21_, the blue shaded volume will expand. The volumes absent of color represent pairwise sets of matrix elements that have no effect on *λ* at the quadratic scale.

**Figure 4:**
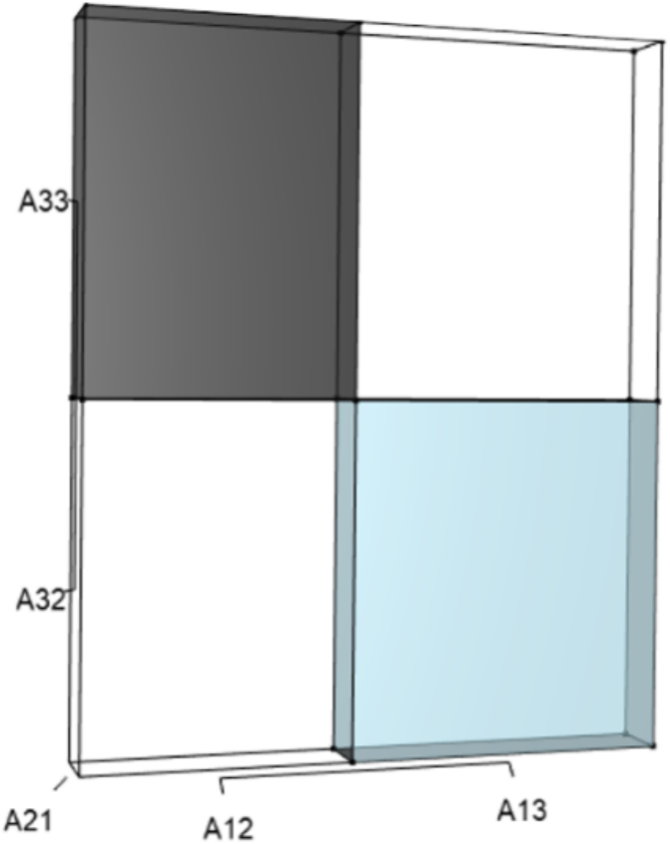
The orthotope of *r* (Eq. (12)). Negative (black) and positive (light blue) contributions exist from cubic interactions among three-way sets of matrix elements (Table 1). Should managers increase *A*_13_, *A*_21_, or *A*_32_, then the light blue volume (*A*_13_*A*_21_*A*_32_) will increase. However, an increase in *A*_21_ would simultaneously expand the black volume (*A*_21_*A*_12_*A*_33_), which functions to counterbalance the blue. The final value of *r* is the net difference after each of these opposing effects have been tabulated, and it is only the net value of *r* that drives changes in final value of *λ*. In the deer example, all other three-way sets of matrix elements have no effect on the characteristic equation at the cubic scale.

Figures 1–4 show the *λ*^3^, *pλ*^2^, *qλ*, and r orthotopes (respectively) in Eq. (13). Each dimension in the orthotopes are scaled to the appropriate *A*_*ij*_ values (as specified in Eq. (11)) and to the *λ* that balances them.

A 10% increase in the survival of early-breeding adults (*A*_32_) gives:

(14) \begin{eqnarray*}A= \left[ \begin{array}{@{}ccc@{}} \displaystyle 0&\displaystyle 0.58&\displaystyle 0.70\\ \displaystyle 0.14&\displaystyle 0&\displaystyle 0\\ \displaystyle 0&\displaystyle 0.84&\displaystyle 0.80 \end{array} \right] ,\end{eqnarray*}

which yields

**Figure 5:**
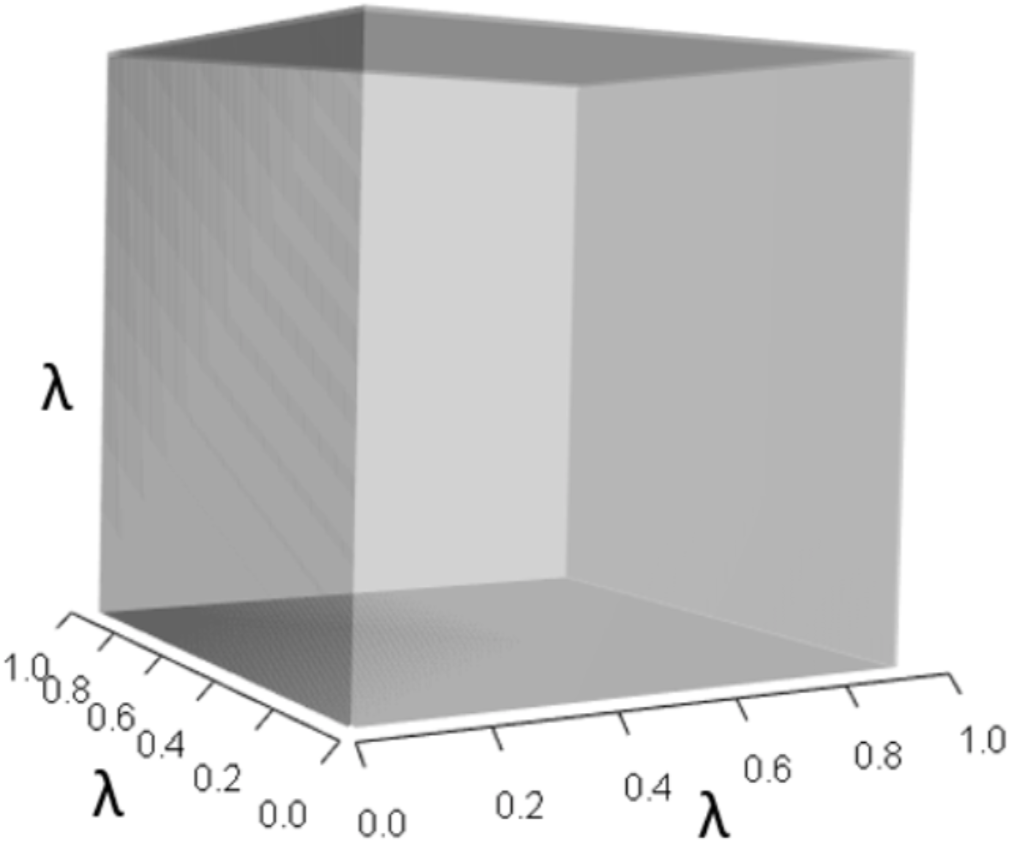
The slight increase in the *λ*^3^ orthotope given the hypothetical 10% increase in *A*_32_. The inner dark grey orthotope shows the volume of the unperturbed system, while the very subtle light grey slivers (on the right and top edges) show the (miniscule) volumetric increase that arose from this 10% alteration to *A*_32_.

**Figure 6:**
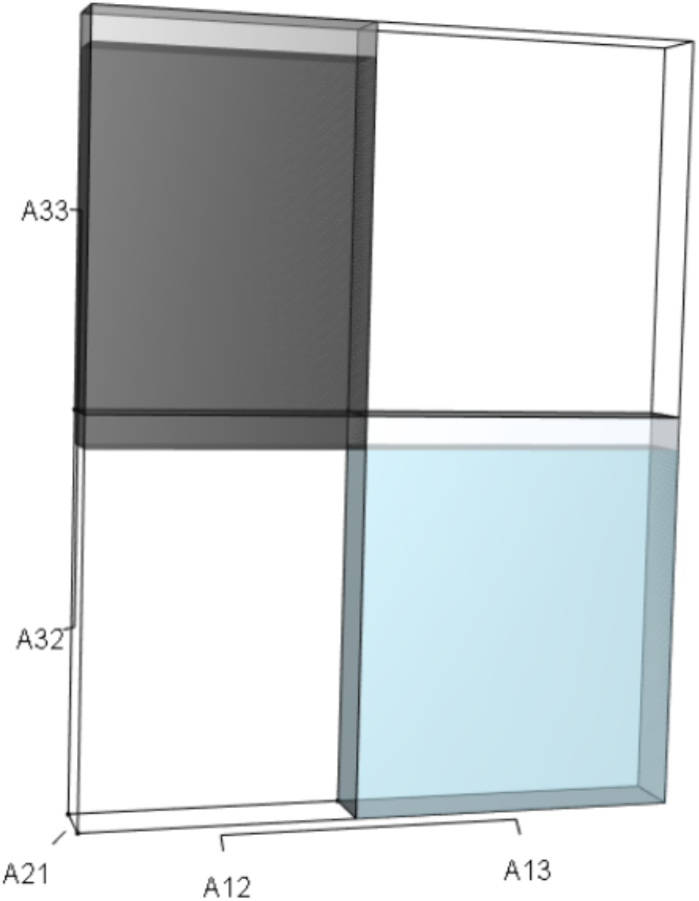
The *r* orthotope given the hypothetical 10% increase in *A*_32_. The black and sky blue volumes represent the volumes of the unperturbed system. The subtle blue sliver (at the top of the blue) shows the increase in the *A*_13_*A*_21_*A*_32_ volume given the 10% increase in *A*_32_. The grey volume shows an upward shift to *A*_21_*A*_12_*A*_33_, but the magnitude of the black volume remains unchanged. The overall increase in the *A*_13_*A*_21_*A*_32_ volume, however, not only depended on the change to *A*_32_, but also on the situational values of *A*_21_ and *A*_13_.

(15) \begin{eqnarray*}{\lambda }^{3}+(-0.8){\lambda }^{2}+(-0.081)\lambda +(-0.0173)=0,\end{eqnarray*}

and a corresponding 1% increase in *λ*. This management strategy alters the characteristic equation by (a) increasing the volume of the *λ*^3^ orthotope (Fig. 5), (b) rescaling the two *λ* dimensions in the *pλ*^2^ orthotope to decrease its overall contribution, (c) rescaling the *λ* dimensions in the trio of *qλ* sub-orthotopes to decrease their overall net contribution, and by (d) decreasing the overall contribution of the *r* orthotope (Fig. 6).

Alternatively, a 10% increase in survival of late-breeding adults (*A*_33_) gives:

(16) \begin{eqnarray*}A= \left[ \begin{array}{@{}ccc@{}} \displaystyle 0&\displaystyle 0.58&\displaystyle 0.70\\ \displaystyle 0.14&\displaystyle 0&\displaystyle 0\\ \displaystyle 0&\displaystyle 0.77&\displaystyle 0.88 \end{array} \right] ,\end{eqnarray*}

which yields

**Figure 7:**
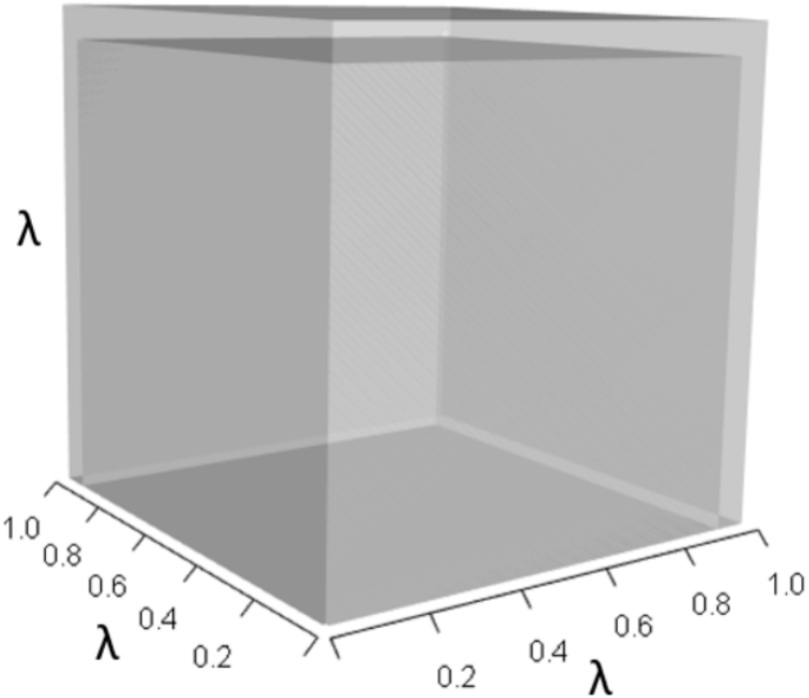
The *λ*^3^ orthotope given the hypothetical 10% increase in *A*_33_. The inner dark grey orthotope shows the volume of the unperturbed system, while the light grey slivers (at the edges) show the volumetric increase that arose from the alteration. Comparison of this orthotope to Fig. 5 makes it clear that *A*_33_ has more influence on *λ* than *A*_32_.

**Figure 8:**
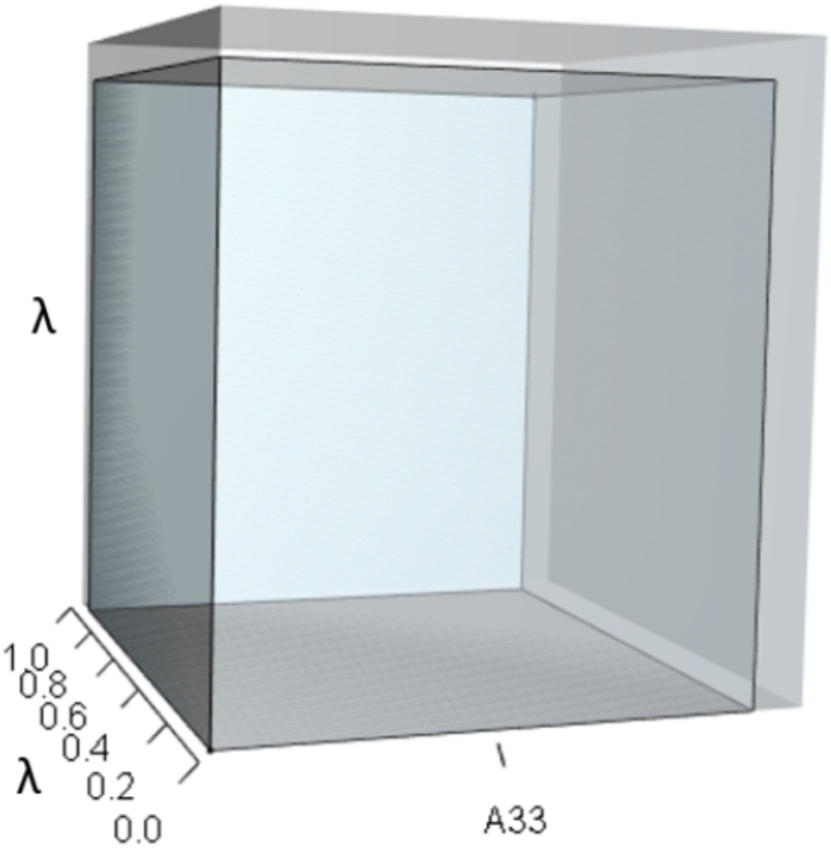
The orthotope of *pλ*^2^ given the hypothetical 10% increase in *A*_33_. The inner sky blue orthotope shows the volume of the unperturbed system, while the subtle blue slivers (at the top and right edges) show the volumetric increase that arose from the alteration of *A*_33_. Notice that both the value of *A*_33_ and the value of *λ* increased.

**Figure 9:**
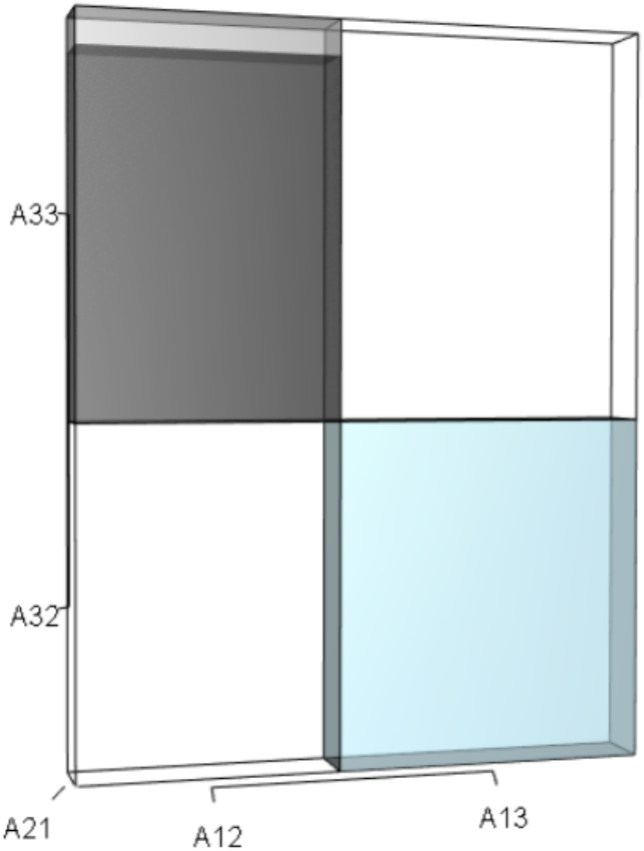
The *r* orthotope given the hypothetical 10% increase in *A*_33_. The black and sky blue represent the volumes in the unperturbed system. The grey sliver shows the increase in the *A*_33_*A*_21_*A*_12_ volume given the 10% increase in *A*_33_. The *A*_32_*A*_21_*A*_13_ volume did not change. The overall increase in the *A*_33_*A*_21_*A*_12_ volume not only depended on the change to *A*_33_, but also on the situational values of *A*_21_ and *A*_12_.

(17) \begin{eqnarray*}{\lambda }^{3}+(-0.88){\lambda }^{2}+(-0.081)\lambda +(0.004)=0,\end{eqnarray*}

which equates to a 7% increase in *λ*. This change alters the characteristic equation by (a) increasing the volume of the *λ*^3^ orthotope (Fig. 7), (b) decreasing the contribution of the *pλ*^2^ orthotope (Fig. 8), (c) rescaling the *λ* dimensions in the trio of *qλ* sub-orthotopes to decrease their overall net contribution, and by (d) increasing the overall contribution of the *r* orthotope (Fig. 9).

**Figure 10:**
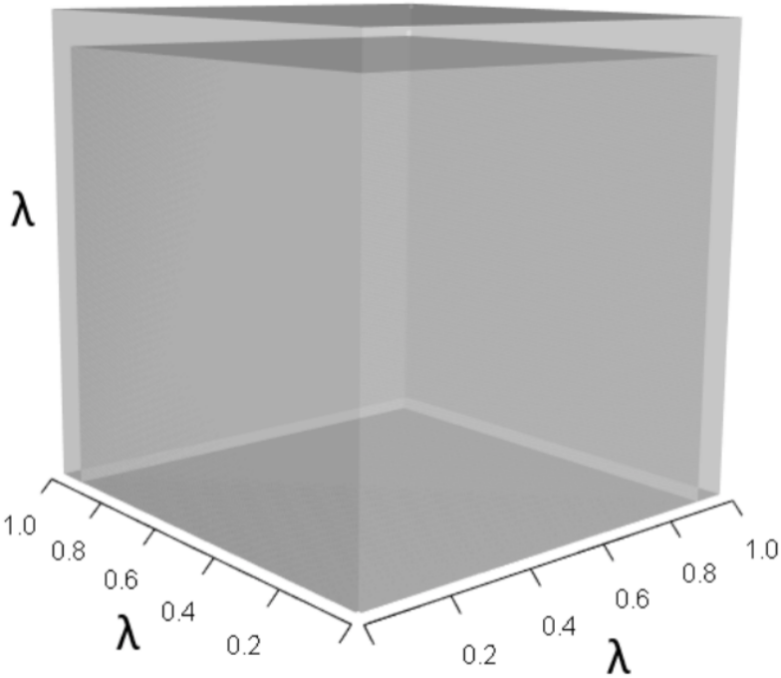
The *λ*^3^ orthotope given the hypothetical 10% increase in *A*_32_ and *A*_33_. The inner dark grey orthotope shows the volume of the unperturbed system, while the light grey segments show the volumetric increase that arose from the simultaneous alterations.

**Figure 11:**
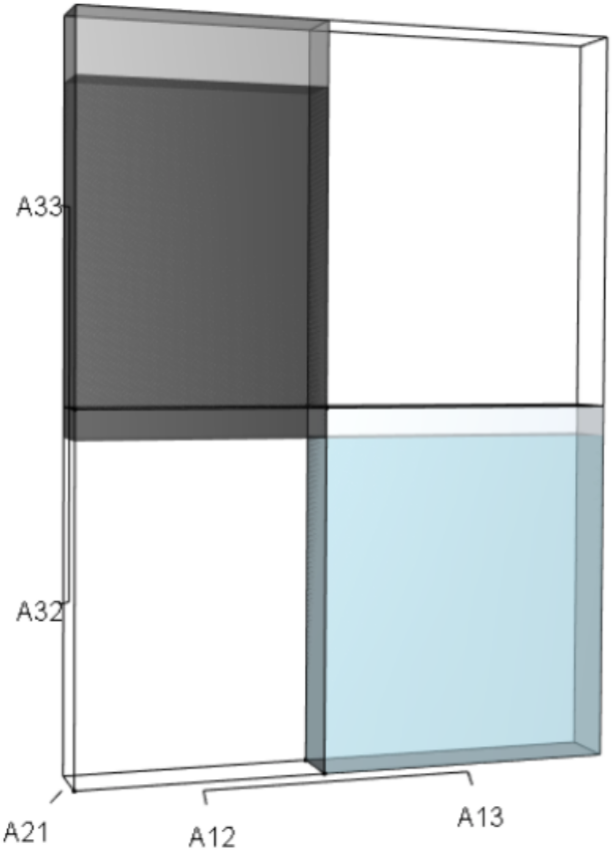
The *r* orthotope given the hypothetical 10% increase in *A*_32_ and *A*_33_. The black and sky blue represent the volumes in the unperturbed system. The subtle blue sliver shows the increase in the *A*_13_*A*_21_*A*_32_ volume given the 10% increase in *A*_32_ and *A*_33_. The grey volume shows both the upward shift and the increase in the *A*_21_*A*_12_*A*_33_ volume. The overall increase in the blue volume (*A*_13_*A*_21_*A*_32_) not only depended on the change to *A*_32_, but also on the situational values of *A*_21_ and *A*_13_. As well, the overall increase in the black volume (*A*_21_*A*_12_*A*_33_) depended on the change in *A*_33_ and on the situational values of *A*_21_ and *A*_12_. Since both volumes increased, and they counteract each other, the net change in *r* was negligible. Thus, any substantial change to the characteristic equation (and therefore *λ*) consequent to the simultaneous increase in *A*_32_ and *A*_33_ would have had to have been governed by net changes in *p* or *q*. Indeed, the alteration of *p* drove the response in *λ* to this targeted management scenario.

Finally, a 10% simultaneous increase in survival of early- and late-breeding adults (*A*_32_, *A*_33_) gives:

(18) \begin{eqnarray*}A= \left[ \begin{array}{@{}ccc@{}} \displaystyle 0&\displaystyle 0.58&\displaystyle 0.70\\ \displaystyle 0.14&\displaystyle 0&\displaystyle 0\\ \displaystyle 0&\displaystyle 0.84&\displaystyle 0.88 \end{array} \right] ,\end{eqnarray*}

(19) \begin{eqnarray*}{\lambda }^{3}+(-0.88){\lambda }^{2}+(-0.081)\lambda +(-0.0108)=0,\end{eqnarray*}

which also equates to a 7% increase in *λ*. The changes alter the characteristic equation by (a) increasing the volume of the *λ*^3^ orthotope (Fig. 10), (b) decreasing the contribution of the *pλ*^2^ orthotope, (c) rescaling the *λ* dimensions in the trio of *qλ* sub-orthotopes to decrease their overall net contribution, and by (d) slightly decreasing the overall contribution of the *r* orthotope (Fig. 11).

## Discussion

The behavior of *λ* regularly defies intuition. But the linkage between a managerial action and its effect on *λ* can become more transparent when cause and effect is thought of as a two-step process. A targeted managerial change to an *A*_*ij*_ first alters the configurations of the *p*, *q*, and *r* orthotopes, then the value of *λ* (and the configuration of the *λ*^3^ orthotope) adjusts to rebalance the overall equation to zero. In this two-step process, changes to the *A*_*ij*_s always alter the configuration of the *p*, *q*, or *r* orthotopes, but only the net changes to these orthotopes alter *λ*. In actuality, the response of *λ* happens instantaneously, but we use this procedural metaphor to help readers of think more deeply about the linkage.

The mathematical configurations of *A*_*ij*_s in Eqs. (6)–(9) are governed by definitions in linear algebra (Strang, 2016), yet the configurations of sums, differences, and products of *A*_*ij*_s suggest that not all *A*_*ij*_s contribute equally to *λ*. Such asymmetries in the configurations of *A*_*ij*_s suggest that targeted alterations to some *A*_*ij*_ are more likely to produce net effects than alterations to others. Indeed, traditional sensitivity analyses (in equation form) have long exposed these differentials (Caswell, 2001). We suggest, however, that IsoPOPd may be used by broader audiences to garner a deeper understanding of why this is so. For example, the deer example illustrated that alterations to an *A*_*ii*_ (that contributes to the *p* orthotope) had higher propensity to influence net changes in the characteristic equation (and therefore *λ*) than the *A*_*ij*_s that contributes only to *r*. Calculations using traditional sensitivity analysis for white-tailed deer corroborate this result; *A*_33_ has a higher influence on *λ* than *A*_32_.

It might seem intuitive that a management strategy designed to improve survival of all individuals would proportionally increase the growth rate. But we just showed that in some life cycles, this intuition may be flawed. The deer demonstration revealed that a management strategy designed to simultaneously increase survival in the 2nd and 3rd stages is no better off than a management strategy designed to increased survival in the 3rd stage only. Due to the asymmetries of the vital rates in Eq. (9), simultaneous modification of *A*_32_ and *A*_33_ differentially altered the characteristic equation at the normal scale, the quadratic scale, and produced two (opposing!) influences at the cubic scale, lending an overall unintuitive (and non-linear) response in *λ*. Indeed, it was the counterbalancing of the positive and negative expansions from *A*_32_ and *A*_33_ in the *r* orthotope that neutralized any additive benefit of increasing *A*_32_ (Fig. 11). This seemingly peculiar result arose from the life history of the deer, and may or may not extend to management in other species. Afterall, sensitivity analysis is situational (De Kroon, Van Groenendael & Ehrlen, 2000).

It is natural to ponder the biological interpretations of *p*, *q*, and *r*, but such a discussion is outside of the scope of this work. Rather, the scope of this demonstration is to provide a general tool for non-specialized audiences to heuristically discover why the behavior of *λ* can defy intuition, and how to leverage the mathematics to their benefit in their strategic planning. Simply stated, management strategies that influence vital rates in the *r* orthotope are not as influential on *λ* as management strategies that influence the *p* or *q* orthotopes. As well, any alteration that counteracts another in magnitude and directionality will result in unchanged net values, which in turn will fail to alter *λ*. Consideration of these mathematical relationships may aid managers in identifying the most efficient intervention strategies for attaining their population goals.

In our demonstration and discussion, we have used the concept of vital rates and matrix elements interchangeably. In the deer example, stage one individuals can die or advance to stage two, stage two individuals can die or advance to stage three, and stage three individuals can die or remain in stage three, so the probability of any transition (given survival) is equal to one. However, in more complicated life histories, transitional matrix elements (*A*_*ij*_ where *i* = 2, 3, and *j* = 1, 2, 3) are defined as the product between survival and transition (Caswell, 2001). This definition becomes important when individuals can transition through their life history in more ways than one. For example, in PMMs where *A*_22_ is non-zero, animals in stage two could (1) die, (2) survive and remain in stage two, or (3) survive and mature into stage three. In this case, the probability of transition among stages two and three must be incorporated into the values of both *A*_22_ and *A*_32_ (and not just in *A*_32_ as we did in our example). Although the compound nature of transition and survival are undoubtedly important in PMMs, we leave it to the user to properly calculate each *A*_*ij*_ for input in the IsoPOPd software.

## Conclusion

Population matrix models are foundational in the study of ecology, population dynamics, wildlife management, and conservation biology (Caswell, 2001), and sensitivity investigations of the relationships between *A*_*ij*_ and *λ* are not novel (see Salguero-Gómez et al., 2014; Salguero-Gómez et al., 2016). However, it is our hope that the IsoPOPd software illuminates to broader audiences the behavior of *λ* in the context of decision making in population management, and how such knowledge can be used as leverage to achieve conservation goals.

##  Supplemental Information

10.7717/peerj.8018/supp-1 Supplemental Information 1 Mathematical derivations

## Additional Information and Declarations

### Data Availability

The following information was supplied regarding data availability: Data is available at Cornell University eCommons: https://doi.org/10.7298/bcmg-7w08.
