# Supplementary material for: Another look at the eigenvalues of a population matrix model
Source: PeerJ. 2019 Nov 11;7:e8018. doi: 10.7717/peerj.8018 (PMC6855200; doi:10.7717/peerj.8018)
Supplement: Supplemental Information 1 [file peerj-07-8018-s001.docx]

SUPPORTING INFORMATION

Hanley. B., P. Connelly, and B. Dennis. 201X. Another look at the eigenvalues of a population matrix model.

**Mathematical derivations**

Given a population matrix representing any 3-stage life history, where the vital rates are arbitrarily denoted $a_{ij}$ for $i=1, 2, 3$, and $j=1, 2, 3$, and of the form:

$\mathbf{L}=\left[ \begin{matrix} a_{11} & a_{12} & a_{13} \\ a_{21} & a_{22} & a_{23} \\ a_{31} & a_{32} & a_{33} \end{matrix} \right]$, (1S)

then the characteristic equation is in the form (Beyer, 1978):

$\det\left( \mathbf{L}-\lambda\mathbf{I} \right)=0$, (2S)

where $\mathbf{I}$ is the $3 \times3$ identity matrix, and $\lambda$ is an eigenvalue of $\mathbf{L}$. Equivalently,

$\lambda^{3}+p\lambda^{2}+q\lambda+r=0$, (3S)

where

$p=(-a_{11}$ ${-a}_{22}-a_{33})$, (4S)

$q=(a_{11}a_{22}$ $+a_{22}a_{33}$ ${+a}_{11}a_{33}$ $-a_{32}a_{23}{-a_{21}a}_{12}$ $-a_{13}a_{31}$), (5S)

and

${r=(a}_{11}a_{32}a_{23}{+a}_{12}a_{21}a_{33}+{a_{31}a}_{13}a_{22}$ $-a_{11}a_{22}a_{33}{-a}_{21}a_{13}a_{32}{-a}_{31}{a_{12}a}_{23}$). (6S)

We refer to $p, q,$ and $r$from eqs. 4S-6S as superparameters (e.g. Hanley & Dennis, 2019). The roots of the characteristic equation are the eigenvalues of the matrix (Cull & Vogt, 1973).

Let $\lambda$ be a real number. Rewriting, eq. 3S, we have:

$\lambda^{3}+(-a_{11}$ ${-a}_{22}-a_{33})\lambda^{2}$

$$+\left( a_{11}a_{22}+a_{22}a_{33}{+a}_{11}a_{33}-a_{32}a_{23}{-a_{21}a}_{12}-a_{13}a_{31} \right)\lambda$$

$+{(a}_{11}a_{32}a_{23}{+a}_{12}a_{21}a_{33}+{a_{31}a}_{13}a_{22}-a_{11}a_{22}a_{33}{-a}_{21}a_{13}a_{32}{-a}_{31}{a_{12}a}_{23}=0.$ (7S)

Collecting positive terms on one side and negative terms on the other, we have:

$$\lambda^{3}+\left( a_{11}a_{22}+a_{22}a_{33}{+a}_{11}a_{33} \right)\lambda+a_{11}a_{32}a_{23}{+a}_{12}a_{21}a_{33}+{a_{31}a}_{13}a_{22}=$$

$(a_{11}$ ${+ a}_{22}+a_{33})\lambda^{2}+\left( a_{32}a_{23}{+a_{21}a}_{12}+a_{13}a_{31} \right)\lambda$

$+a_{11}a_{22}a_{33}{+a}_{21}a_{13}a_{32}{+a}_{31}{a_{12}a}_{23}$, (8S)

which leads to positive and negative contributions of the balance equation (see Table 1).

Now let $\lambda$ be a complex number, where $\lambda=g+hi.$ The characteristic equation for a 3-stage population matrix model is then:

${(g+hi)}^{3}+p{(g+hi)}^{2}+q(g+hi)+r=0$, (9S)

Where p, q, and are as in eqs. 4S-6S. Rewriting eqs. 3S, we have:

${(g+hi)}^{3}+(-a_{11}$ ${-a}_{22}-a_{33})({g+hi)}^{2}$

$$+\left( a_{11}a_{22}+a_{22}a_{33}{+a}_{11}a_{33}-a_{32}a_{23}{-a_{21}a}_{12}-a_{13}a_{31} \right)(g+hi)$$

$+{(a}_{11}a_{32}a_{23}{+a}_{12}a_{21}a_{33}+{a_{31}a}_{13}a_{22}-a_{11}a_{22}a_{33}{-a}_{21}a_{13}a_{32}{-a}_{31}{a_{12}a}_{23}=0.$ (10S)

Expanding, we have:

$$g^{3}+3g^{2}hi-3gh^{2}-h^{3}i$$

$+(-a_{11}$ ${-a}_{22}-a_{33})(g^{2}+2ghi-h^{2})$

$$+\left( a_{11}a_{22}+a_{22}a_{33}{+a}_{11}a_{33}-a_{32}a_{23}{-a_{21}a}_{12}-a_{13}a_{31} \right)(g+hi)$$

$+{(a}_{11}a_{32}a_{23}{+a}_{12}a_{21}a_{33}+{a_{31}a}_{13}a_{22}-a_{11}a_{22}a_{33}{-a}_{21}a_{13}a_{32}{-a}_{31}{a_{12}a}_{23}=0.$ (11S)

Further:

$$g^{3}+3g^{2}hi-3gh^{2}-h^{3}i$$

$-a_{11}g^{2}$ ${-a}_{22}g^{2}-a_{33}g^{2} -a_{11}2ghi$ ${-a}_{22}2ghi-a_{33}2ghi +a_{11}h^{2}$ ${+a}_{22}h^{2}+a_{33}h^{2}$

$$+a_{11}a_{22}g+a_{22}a_{33}g{+a}_{11}a_{33}g-a_{32}a_{23}g{-a_{21}a}_{12}g-a_{13}a_{31}g$$

$${+a}_{11}a_{22}hi+a_{22}a_{33}hi{+a}_{11}a_{33}hi-a_{32}a_{23}hi{-a_{21}a}_{12}hi-a_{13}a_{31}hi$$

$+a_{11}a_{32}a_{23}{+a}_{12}a_{21}a_{33}+{a_{31}a}_{13}a_{22}-a_{11}a_{22}a_{33}{-a}_{21}a_{13}a_{32}{-a}_{31}{a_{12}a}_{23}=0.$ (12S)

Collecting positive terms on one side and negative terms on the other, we have:

$g^{3}+3g^{2}hi+a_{11}h^{2}$ ${+a}_{22}h^{2}+a_{33}h^{2}+a_{11}a_{22}g+a_{22}a_{33}g{+a}_{11}a_{33}g$

$${+a}_{11}a_{22}hi+a_{22}a_{33}hi{+a}_{11}a_{33}hi+a_{11}a_{32}a_{23}{+a}_{12}a_{21}a_{33}+{a_{31}a}_{13}a_{22}$$

$=3gh^{2}+h^{3}i+a_{11}g^{2}$ ${+a}_{22}g^{2}+a_{33}g^{2}+a_{11}2ghi$ ${+a}_{22}2ghi+a_{33}2ghi$

$$+a_{32}a_{23}g{+a_{21}a}_{12}g+a_{13}a_{31}g+a_{32}a_{23}hi{+a_{21}a}_{12}hi+a_{13}a_{31}hi$$

$+a_{11}a_{22}a_{33}{+a}_{21}a_{13}a_{32}{+a}_{31}{a_{12}a}_{23}$. (13S)

Collecting real and imaginary components, we have:

$(g^{3}+a_{11}h^{2}$ ${+a}_{22}h^{2}+a_{33}h^{2}+a_{11}a_{22}g+a_{22}a_{33}g{+a}_{11}a_{33}g+a_{11}a_{32}a_{23}{+a}_{12}a_{21}a_{33}+{a_{31}a}_{13}a_{22})$+($3g^{2}h{+a}_{11}a_{22}h+a_{22}a_{33}h{+a}_{11}a_{33}h)$

=($3gh^{2}+a_{11}g^{2}$ ${+a}_{22}g^{2}+a_{33}g^{2}$ $+a_{32}a_{23}g{+a_{21}a}_{12}g+a_{13}a_{31}g+a_{11}a_{22}a_{33}{+a}_{21}a_{13}a_{32}{+a}_{31}{a_{12}a}_{23}$)+($h^{3}+a_{11}2gh$ ${+a}_{22}2gh+a_{33}2gh +a_{32}a_{23}h{+a_{21}a}_{12}h+a_{13}a_{31}h)i$. (14S)

This implies that:

$g^{3}+a_{11}h^{2}$ ${+a}_{22}h^{2}+a_{33}h^{2}+a_{11}a_{22}g+a_{22}a_{33}g{+a}_{11}a_{33}g$

$$+a_{11}a_{32}a_{23}{+a}_{12}a_{21}a_{33}+{a_{31}a}_{13}a_{22}$$

$=3gh^{2}+a_{11}g^{2}$ ${+a}_{22}g^{2}+a_{33}g^{2}$ $+a_{32}a_{23}g{+a_{21}a}_{12}g+a_{13}a_{31}g$

$+a_{11}a_{22}a_{33}{+a}_{21}a_{13}a_{32}{+a}_{31}{a_{12}a}_{23}$, (15S)

and

$3g^{2}{+a}_{11}a_{22}+a_{22}a_{33}{+a}_{11}a_{33}$

$=h^{2}+a_{11}2g$ ${+a}_{22}2g+a_{33}2g +a_{32}a_{23}{+a_{21}a}_{12}+a_{13}a_{31}$. (16S)

Equations 15S-16S leads to two sets of positive and negative contributions of the balance equation (see Tables S.1 and S.2).

| Components of the characteristic balance equation for an  imaginary dominant eigenvalue | |
| --- | --- |
| Negative contribution for the real component ($g$) | Positive contribution for the real component ($g$) |
| $(3g)h^{2}$ | $g^{3}$ |
| $(a_{11}$ ${+a}_{22}+a_{33})g^{2}$ | $\left( a_{11}+a_{22}+a_{33} \right)h^{2}$ |
| ${(a}_{32}a_{23}{+a_{21}a}_{12}+a_{13}a_{31})g$ | ${(a}_{11}a_{22}+a_{22}a_{33}{+a}_{11}a_{33})g$ |
| $(a_{11}a_{22}a_{33}{+a}_{21}a_{13}a_{32}{+a}_{31}{a_{12}a}_{23}$) | ${(a}_{11}a_{32}a_{23}{+a}_{12}a_{21}a_{33}+{a_{31}a}_{13}a_{22})$ |

Table S.1. The positive and negative contributions to the real component of the balance equation for a complex dominant eigenvalue. Here, $g$ is the term that must vary to balance these components to zero.

| Components of the characteristic balance equation for an  imaginary dominant eigenvalue | |
| --- | --- |
| Negative contribution for the imaginary component (*h*) | Positive contribution for the imaginary component ($h$) |
| $h^{2}$ | $3g^{2}$ |
| $(a_{11}$ ${+a}_{22}+a_{33})2g$ | ${(a}_{11}a_{22}+a_{22}a_{33}{+a}_{11}a_{33}$) |
| ${(a}_{32}a_{23}{+a_{21}a}_{12}+a_{13}a_{31})g$ |  |

Table. S.2. The positive and negative contributions to the imaginary component of the balance equation for a complex dominant eigenvalue. Here, $h$ is the term that must vary to balance these components to zero.
